# Electroconvulsive Therapy in a Patient with Dissociative Episodes: A Rare Case Suggesting a Novel Therapeutic Potential

**DOI:** 10.1192/j.eurpsy.2026.11760

**Published:** 2026-07-31

**Authors:** E. B. Avaner, B. Koparal

**Affiliations:** Department of Psychiatry, Gazi University, Ankara, Türkiye

## Abstract

**Introduction:**

Electroconvulsive therapy (ECT) is a well-known intervention for treatment resistant mood disorders but not indicated for dissociative disorders. To date, only few case reports have described incidental improvements in dissociative symptomatology following ECT administered for comorbid depression. Emerging neuroimaging evidence suggests that ECT modulates large-scale brain networks which includes the fronto-limbic system, anterior cingulate cortex and default mode network that overlap with the neural mechanisms implicated in depersonalization and derealization. This overlap suggests that ECT may indirectly facilitate reintegration of self-referential processes through network-level reorganization.

**Objectives:**

To explore the potential therapeutic impact of ECT on dissociative symptomatology through the clinical course of a patient with recurrent episodic dissociative symptoms and comorbid depressive symptoms resistant to pharmacotherapy.

**Methods:**

A 36-year-old female patient was admitted to our inpatient clinic with severe anxiety and episodic dissociative symptoms characterized by amnesia, depersonalization and behavioral disinhibition occurring up to several times a day. Her history was revealed that she had been experiencing dissociative symptoms for approximately ten years,during which she had undergone multiple psychiatric evaluations and received adequate pharmacological and psychotherapautic interventions, with minimal or no clinical improvement. Given the chronic course and resistance to treatments, electroconvulsive therapy under general anesthesia was planned.

**Results:**

The patient underwent six sessions of ECT under close monitoring. During the post-ECT recovery phase, no complications were observed except for transient dissociative symptoms and amnesia. Regular psychiatric evaluations were conducted throughout hospitalization. Pharmacotherapy was adjusted to quetiapine 600 mg/day, fluoxetine 40 mg/day. Frequency and intensity of dissociative symptoms decreased significantly. She also demonstrated a notable improvement in anxiety and irritability. She was discharged from hospital in clinical partial remisssion. The patient was lost to follow-up therefore her long-term clinical course remains unknown.

**Conclusions:**

This case represents one of the few reported instances of dissociative psychopathology demonstrating response to ECT. The observed remission may indicate a restoration of connectivity balance between cortical networks, enhancing integration between affective and self-referential systems. Although preliminary, this case suggest that the therapeutic effects of ECT may extend beyond mood regulation to encompass network-level reintegration in disorders of self. Further systematic studies are needed to clarify indications, underlying mechanisms and ethical implications of ECT as a potential treatment option for severe and refractory dissociative disorders.

**Disclosure of Interest:**

None Declared

